# Design and quantitative evaluation of ‘Aerosol Bio-Containment Device (ABCD)’ for reducing aerosol exposure during infectious aerosol-generating events

**DOI:** 10.1371/journal.pone.0272716

**Published:** 2023-01-06

**Authors:** Michael S. Waring, L. James Lo, Michael A. Kohanski, Elizabeth Kahle, Ian M. Marcus, Heather Smith, Kara L. Spiller, Sharon L. Walker

**Affiliations:** 1 Department of Civil, Architectural and Environmental Engineering, Drexel University, Philadelphia, PA, United States of America; 2 Division of Rhinology, Department of Otorhinolaryngology-Head and Neck Surgery, Perelman School of Medicine, University of Pennsylvania, Philadelphia, PA, United States of America; 3 School of Biomedical Engineering, Science, and Health Systems, Drexel University, Philadelphia, PA, United States of America; 4 Life Sciences Department, Riverside City College, Riverside, CA, United States of America; Beni Suef University Faculty of Veterinary Medicine, EGYPT

## Abstract

The Coronavirus Disease 2019 (COVID-19) pandemic renewed interest in infectious aerosols and reducing risk of airborne respiratory pathogen transmission, prompting development of devices to protect healthcare workers during airway procedures. However, there are no standard methods for assessing the efficacy of particle containment with these protective devices. We designed and built an aerosol bio-containment device (ABCD) to contain and remove aerosol via an external suction system and tested the aerosol containment of the device in an environmental chamber using a novel, quantitative assessment method. The ABCD exhibited a strong ability to control aerosol exposure in experimental and computational fluid dynamic (CFD) simulated scenarios with appropriate suction use and maintenance of device seals. Using a log-risk-reduction framework, we assessed device containment efficacy and showed that, when combined with other protective equipment, the ABCD can significantly reduce airborne clinical exposure. We propose this type of quantitative analysis serves as a basis for rating efficacy of aerosol protective enclosures.

## Introduction

The novel Coronavirus disease 2019 (COVID-19) caused by Severe Acute Respiratory Syndrome Coronavirus 2 (SARS-CoV-2) has an expansive array of respiratory and systemic manifestations [1–3]. COVID-19 transmission occurs following exhalation or release of liquid particles (on a size continuum ranging from fast-settling droplets to persistent airborne aerosols [4–6]) containing SARS-CoV-2 from respiratory tracts of infected individuals. Subsequent viral transmission can occur due to contact with viral-laden surfaces and self-inoculation of the mouth or nose; impact on the face or inhalation into the upper-airway of droplets; or inhalation of aerosols into all regions of the respiratory tract (upper-airway, trachea, and lungs) [7]. Early in the pandemic, there was controversy regarding the role of aerosol inhalation (also called airborne transmission). However, airborne transmission has been recognized by the Centers for Disease Control and Prevention (CDC) [8] and World Health Organization (WHO) [9] as a major source of transmission and may in fact be the dominant transmission pathway in indoor environments [10, 11]. Approximately 14% of adults contracting COVID-19 are hospitalized [12], and of that group, 9.7% to 16% require mechanical ventilation, which involves high risk airway procedures such as intubation [13, 14]. Hospitalization and intensive care unit (ICU) admission rates with the delta variant of SARS-CoV-2 are likely increased relative to earlier variants of SARS-CoV-2; however, the relative impact of this variant is still under investigation at this time.

There is evidence from the SARS and Middle East Respiratory Syndrome (MERS) outbreaks, as well as emerging evidence from the COVID-19 pandemic, that frontline healthcare workers, who are also likely to participate in high risk aerosol generating procedures (AGPs), are at risk for contracting viral respiratory diseases [7, 15–19]. AGPs are defined as procedures with potential to generate infectious respiratory particles at higher concentrations than breathing, talking, coughing, or sneezing, or procedures that create uncontrolled respiratory secretions [20]. AGPs can include procedures that are medically necessary to treat those with moderate or severe COVID-19, encompassing intubation, extubation, non-invasive positive pressure support (CPAP, BiPAP), delivering nebulizer treatments, and delivery of oxygen over 6 L/min [20]. While there is emerging data that the specific instrumentation associated with some AGPs may not generate particles above physiologic baseline [21, 22], these types of procedures often require prolonged close contact with infected individuals and can be associated with production of uncontrolled respiratory secretions or significant coughing in those suffering from COVID-19. These clinical interactions are considered high risk events, since the proximity to an aerosol or droplet source increases the risk of exposure and successful viral transmission, particularly as the distance between particle source (e.g., airway during an expiratory or aerosol-generating event) and susceptible host decreases to less than 1 m in separation [23], a typical situation during most clinical interactions.

With a dearth of appropriate personal protective equipment (PPE) in the early days of the COVID-19 pandemic, these concerns motivated new efforts to develop devices to protect healthcare workers during AGPs such as intubation [24–29]. There are no U.S. Food and Drug Administration (FDA) fully authorized devices designed for protection from aerosols during AGPs, and there are also no standards for rating or assessing the efficiency of particle containment or removal with use of these types of protective containment devices, which do exist for masks and respirators. Additionally, emerging evidence from case studies demonstrate that protective barrier devices can even concentrate airborne particles and increase the risk of aerosol exposure [25]. These findings underscore the need for an easily and rapidly deployable aerosol bio-containment device that allows safe removal of aerosolized viral particles without contaminating the room. Moreover, the emerging ubiquity of “one-off” protective barrier devices [28] highlights the need to establish a framework for a comprehensive assessment of aerosol capture efficiency with such devices.

We designed and built an aerosol bio-containment device (ABCD) to contain and remove expelled aerosol (and droplets) while allowing healthcare workers to perform airway procedures. Here, we investigate the ability of the ABCD to contain aerosols across a range of utilization scenarios, using a novel, quantitative assessment test. Experimental results were used to tune to computational fluid dynamic (CFD) simulations, and then CFD was used to extrapolate ABCD effectiveness to larger emitted particle sizes than the test aerosol. Using a log-risk-reduction protection framework, we assessed the device containment efficacy to understand how, when combined with other PPE, the ABCD has the potential to significantly reduce airborne clinical exposure. We propose that this type of analysis can serve as a basis for assessment of aerosol protective enclosures.

## Materials and methods

### ABCD design

**Fig 1:**
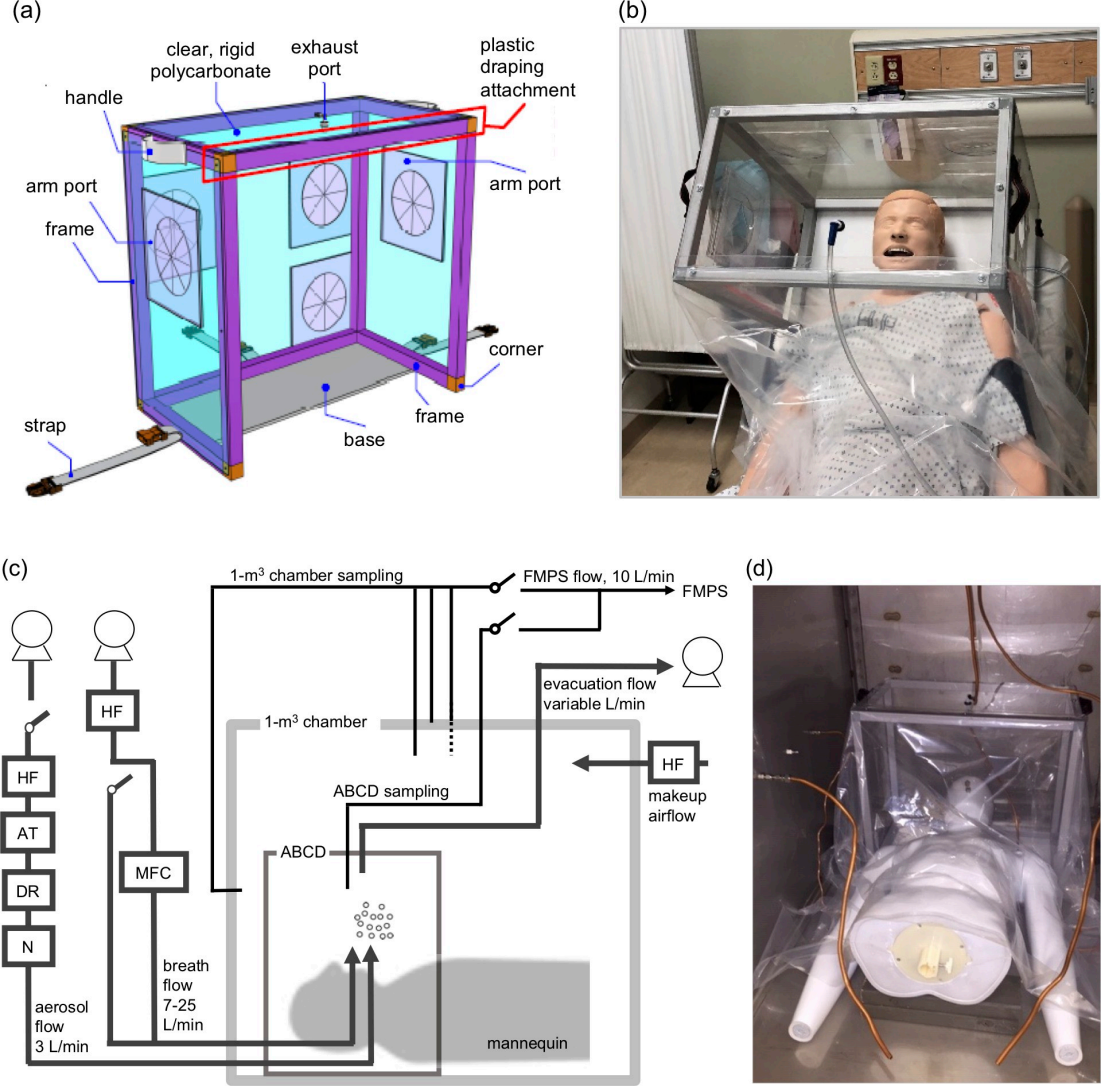
ABCD design and experimental apparatus. **(a)** Schematic of aerosol bio-containment device (ABCD) and **(b)** photograph of ABCD in a simulated clinical patient scenario. The ABCD has a volume of 0.1 m^3^ and measures 0.52 m high, 0.58 m wide, and 0.34 m deep, and has multiple straps so it can be secured to a bed at different angles. **(c)** Schematic of 1 m^3^ stainless steel, environmental chamber system for aerosol evaluation testing, and **(d)** photograph of mannequin and ABCD within the chamber. Chamber system abbreviations; HF = HEPA filter; AT = atomizer; DR = diffusion drier; N = neutralizer; MFC = mass flow controller; FMPS = TSI Fast Mobility Particle Sizer 3091, which measures particles in range of 0.0056 to 0.56 μm in size-resolved basis over 32 bins at a time-resolution of 1 s.

The aerosol bio-containment device (ABCD), as shown in Fig 1A, was designed to isolate a patient’s head and top of the shoulders, as shown in Fig 1B, to contain and remove expelled aerosol using external suction tubing connected to an exhaust port, while healthcare workers perform airway procedures. The ABCD has dimensions of 0.52 m height, 0.58 m width, and 0.34 m depth, for a total volume of 0.1 m^3^. It is composed of an aluminum frame, with attached rigid and transparent polycarbonate sheets on the top, back, and left and right sides. There are flexible layered plastic covered ports at the head of the patient for the primary proceduralist and side ports for an assistant, as well as a flexible plastic drape in the front, and a dedicated port for lines or wires associated with equipment needed for AGPs. The ABCD has an evacuation suction port at the top, which connects to standard 0.25 in. hospital or other suction tubing that can be attached to a wall suction or portable pump system to safely clear aerosol from the ABCD to minimize clinical worker exposures. The device has a partial base, with straps to secure it to hospital bed, stretcher, or operating room table with a patient in a supine or semi-upright position. It has two handles and weighs 4 kg (9 lbs.) for easy transport.

This final design of the ABCD, including its size and materials, the number and location of each port, and the detachable front drape, was fixed after several iterations to maximize the mobility of the proceduralist(s), to minimize set up time, and to allow for sterilization and re-use. Besides the aerosol containment effectiveness study presented here, the usability of the ABCD is being evaluated in a clinical trial (NCT04532112) assessing the feasibility of the ABCD in a clinical setting, which will assess issues such as ergonomics and usability when performing airway procedures, procedures and protocols for device storage and cleaning, among others.

### Experimental design

#### ABCD testing apparatus

The effectiveness of the ABCD for aerosol containment was evaluated in a laboratory setting using a 1 m^3^ stainless steel, environmental chamber system shown schematically in Fig 1C, under different test conditions. The ABCD itself was placed inside the 1 m^3^ environmental chamber, over the top of a mannequin used to represent a patient, as shown in Fig 1D.

To simulate a patient aerosol emission, sodium chloride (NaCl) aerosols were emitted into the ABCD through a tube that penetrated through the mannequin’s mouth region, using an aerosol generation system of a pump, filter, atomizer with NaCl solution in deionized water (TSI Aerosol Generator 3076), diffusion dryer (TSI Diffusion Dryer 3062), and neutralizer (TSI Aerosol Neutralizer 3077A) in series. The aerosol flow rate was controlled with a critical orifice as part of the atomizer and was 3 L/min, so a breathing makeup airstream consisting of a separate pump, filter, and mass flow controller was used to provide additional flow at 7 L/min out of a second tube from the mannequin’s mouth region, allowing a total breathing flow of 10 L/min to be achieved. At certain points in each experiment, an expiratory event of larger flow, such as that which might be seen in a coughing event, was simulated. To do so, the mass flow controller was temporarily bypassed using a switching arrangement, so that ~25 L/min rather than 7 L/min was delivered the breathing makeup airstream.

Aerosol concentrations could be measured directly either in the 1 m^3^ environmental chamber or in the ABCD, depending on the phase of the experiment, using a Fast Mobility Particle Sizer (TSI FMPS 3091), which measures aerosols in the range of 0.0056 to 0.56 μm over 32 bins at a time-resolution of 1 s, at a flow rate of 10 L/min. Aerosol sampling occurred either in the 1 m^3^ chamber via four ports sampling uniformly around the back and sides of the chamber in a combined airstream, or in the ABCD itself at a sampling location about 2.5 cm from the ABCD exhaust port. Since outflow due to evacuation and FMPS flows were greater than the aerosol and breath inflows into the ABCD, different amounts of makeup air depending on the experiment (based on total flow balancing) entered the 1 m^3^ environmental chamber through HEPA filtration. Aerosol number and mass concentrations were both used for analysis, in case certain trends manifested only in either. For all analysis, an aerosol diameter cutoff of >0.05 μm was enforced, since viral containing particles of interest are larger [30].

#### ABCD testing protocol

The performance of the ABCD was evaluated at different evacuation flow rates and arrangements of openings of the ABCD seals and drape. Different evacuation flows were accomplished by using one (“Low”), two (“Med”), or three (“High”) vacuum pumps. Arm port seals were varied as fully sealed (“Closed”), tightly sealed around mannequin hands (“Hands”), or held open with tape (“Open”) to investigate how use of the arm ports by proceduralists might influence aerosol containment by the ABCD. The patient drape was varied as pressed firmly around the mannequin torso (“Neat”), with a 7.5 cm gap around the torso (“Gap”), or not present at all (“Absent”).

**Fig 2:**
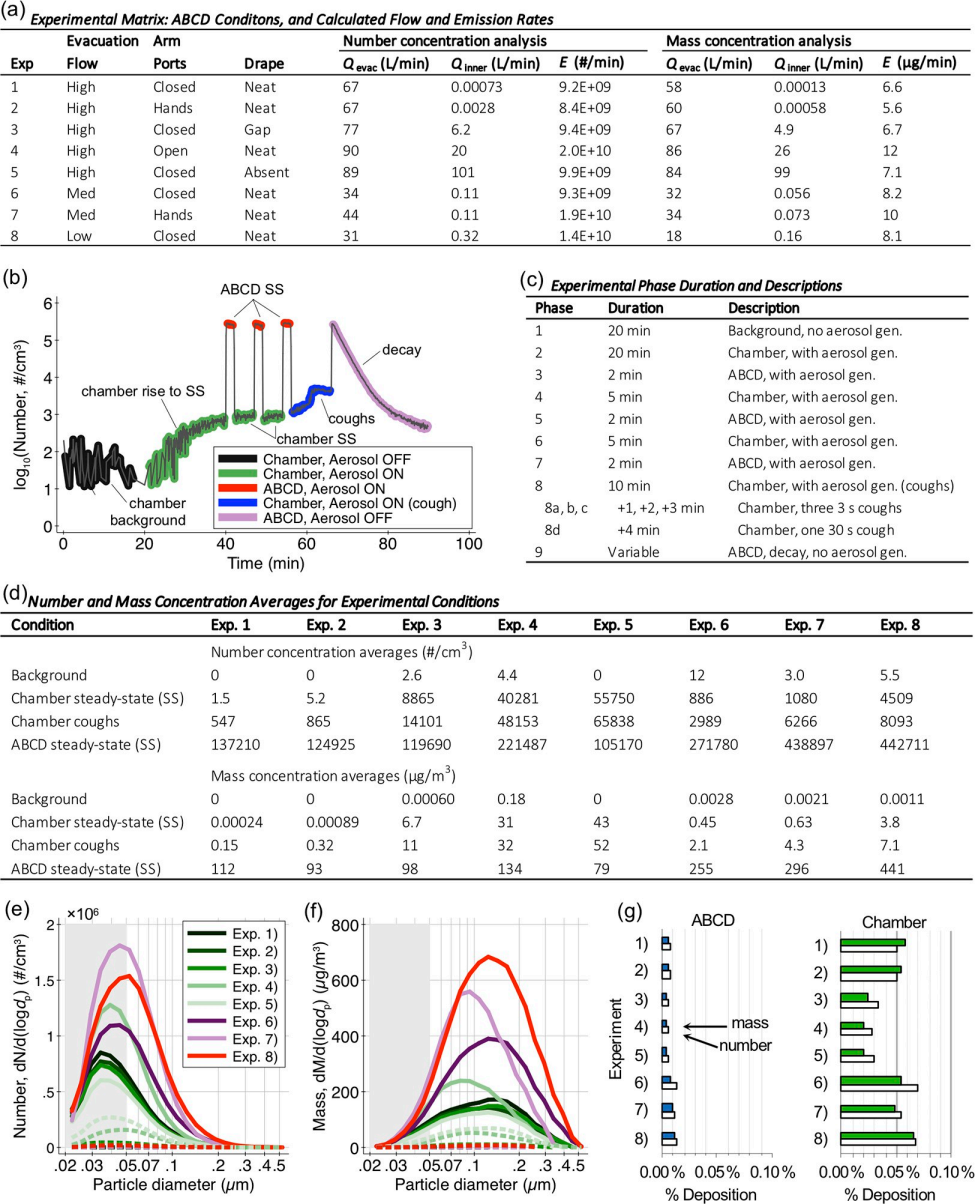
ABCD experimental conditions and summary. **(a)** Description of experimental test conditions and fitted flow and emission rates; **(b)** time series of typical experimental measurements (using Exp. 6 as an example); **(c)** description of experimental phases corresponding to time series in (b); **(d)** average number and mass concentrations in major experimental phase groupings; average **(e)** size and **(f)** mass distributions in ABCD and 1 m^3^ environmental chamber during steady state phases; **(g)** estimate of percentage of aerosol lost to deposition in ABCD and environmental chamber during steady state phases.

Eight experiments with different combinations were conducted, with the matrix as shown in Fig 2A. In experiments 1 to 5, High evacuation flows were used, but with varying degrees of sealing for the arm ports and drape, to demonstrate the importance of the seals on aerosol containment. In experiments 6 to 8, Med or Low evacuation flows were used, but with good closure for the ports and draping, to determine how changing the evacuation flow influenced containment. Evacuation flows were not measured directly, but they were estimated using measured number or mass concentrations and data-fitting techniques (see Data Analysis section below and Supporting Information for more information), and these ranged from 58 to 90 L/min for High flow cases, 32 to 44 L/min for Med flow cases, and 18 to 31 L/min for Low flow cases (see Fig 2A).

For all eight experiments, the same test procedure was followed, which consisted of nine phases as demonstrated in Fig 2B and 2C. Phase 1 established a background and showed that filtered air in the 1 m^3^ environmental chamber had negligible aerosol concentrations prior to intentional generation. In Phase 2, activation of the aerosol generation system resulted in increasing aerosol concentrations in the ABCD and some aerosol escaping the ABCD, with the magnitude of aerosol escaping determined by the combination of test conditions. Phase 2 occurred for 20 min, which was long enough for concentrations in the ABCD and environmental chamber to reach a steady state (denoted SS on plot). With steady concentrations established, the next set of phases alternated sampling between the ABCD (Phases 3, 5, and 7) and 1 m^3^ environmental chamber (Phases 4 and 6) to demonstrate the containment ability of the ABCD for each test condition. Phase 8 challenged the ABCD’s ability more strongly, and it consisted of four pseudo-coughing events that were simulated by increasing the makeup breath airstream from 7 to ~25 L/min. Coughing flow rates are highly variable [31], so this reasonable value was set by the supplemental pump maximum flow rate. Three coughing events were short in duration (3 s) and occurred 1 min, 2 min, or 3 min after the start of Phase 8, while the fourth was 30 s in duration and occurred 4 min after the phase start. Finally, during Phase 9, aerosol generation was deactivated, and this phase demonstrated the time required to clear the ABCD of the previously emitted aerosol under active chamber evacuation.

### Data analysis

#### Aerosol number-to-mass conversion and deposition estimations

Analyses in this study used concentration data either as total number and mass concentrations or size-resolved number and mass concentrations. The FMPS directly measures the time- and size-resolved particle number concentrations over 32 bins, which have variable sizes in the measurement diameter range (0.0056 to 0.56 μm). Converting the FMPS size-resolved number counts to total number is calculated by summing over the concentrations in all bins. Size-resolved mass concentrations are calculated by multiplying the size-resolved number concentration by both the spherical volume of particles in each size bin and the density of sodium chloride (2.16 g/cm^3^). Finally, converting the size-resolved mass concentrations to total mass concentration was performed by summing mass concentrations in all bins.

To determine whether deposition of aerosol in the ABCD and 1 m^3^ environmental chamber were meaningful losses as compared to the evacuation losses, the size and integrated number, as well as mass deposition losses were estimated using the theory of Lai and Nazaroff [32]. This theory estimates size resolved deposition rates based on certain inputs, taken from our experimental parameters, including the deposition surface area and volume, aerosol density, and air temperature and (estimated) turbulence characteristics. The size resolved rates were used to compute integrated deposition rates using weighted averages based on size resolved number and mass concentrations in each experiment.

#### Estimating flow rates and aerosol emission rates

Certain parameters were not measured in the experiments due to laboratory measurement constraints but were instead computing using two similar but separate fitting process with data from different experimental phases. These parameters included the (*i*) ABCD evacuation flow rate, (*ii*) escape flow rate from ABCD to 1 m^3^ chamber, and (*iii*) aerosol emission rates. These parameters were used to constrain performance of the ABCD (i.e., calculate containment ability), as well as to set boundary conditions for computational fluid dynamics (CFD) simulations. These fitting processes are briefly described here, with full detail in the Supporting Information.

The first fitting process determined evacuation flow rates, *Q*_evac_ (L/min). Vacuum pumps provided evacuation airflows for the ABCD between ~20 and 90 L/min, depending on the experimental condition (i.e., Low, Med, or High evacuation,). Evacuation flow rates were computed using aerosol concentration data from the Phase 9 transient decay portion of each experiment using standard mass balances to fit the rate of evacuation loss that would yield the observed decay rate in the ABCD. The second fitting process used data from Phases 4 and 6 when the aerosol emission was operating and steady-state particle escape from the ABCD was measured in the 1 m^3^ environmental chamber. Estimated parameters were number or mass emission rate, *E* (#/min or μg/min), and the escape airflow, *Q*_escape_ (L/min), which parameterizes airflow between the ABCD and 1 m^3^ environmental chamber during experiments. This parameter *Q*_escape_ is higher in experiments when relatively more aerosol escapes the ABCD. It is a parameterization that is intended to describe inter-chamber airflow from an average perspective. In a real situation, this airflow would be the driver of aerosol escaping from the ABCD into a room.

#### ABCD performance determinations

The experimental performance of the ABCD was quantified using two metrics: the (*i*) aerosol containment fraction of the device, as well as the (*ii*) airflow-estimated removal efficiency describing how well the ABCD evacuates aerosol and prevents it from escaping into the larger 1 m^3^ chamber.

The first metric is the aerosol containment fraction, as shown in Eq 1:

$$f_{ABCD}=1-\frac{C_{chamber}∙V_{chamber}}{C_{ABCD}∙V_{ABCD}+C_{chamber}∙V_{chamber}}$$ (1)

where *f*_ABCD_ (-) is the aerosol containment fraction of the ABCD; *C*_ABCD_ and *C*_chamber_ (#/cm^3^ or μg/m^3^) are number or mass aerosol concentrations in the ABCD and 1 m^3^ chamber, respectively; and *V*_ABCD_ (m^3^) and *V*_chamber_ (m^3^) are the ABCD and chamber volumes, respectively. The aerosol containment fraction was computed using both number and mass concentrations, using averages at steady state and averages during the simulated coughing event. This containment fraction is useful to provide an understanding of how much of the aerosol would be contained in the ABCD versus a surrounding room at any instant in time.

It is important to note that the volumes, *V*_chamber_ and *V*_ABCD_, using in Eq 1 are not the nominal environmental chamber (1 m^3^) or ABCD (0.1 m^3^) volumes, since those are volumes without any material inside. Instead, these *V*_chamber_ and *V*_ABCD_ are the actual air volumes in each space, accounting for the various materials within each, including the ABCD and lower part of the mannequin body in the 1 m^3^ chamber and the mannequin head and shoulders in the ABCD. The volumes were estimated using known dimensions and volume displacement methods for the mannequin elements, resulting in best estimates of *V*_chamber_ = 0.87 m^3^ and *V*_ABCD_ = 0.093 m^3^.

The second ABCD performance metric did not use measured aerosol concentrations, but instead used the fitted values of the evacuation and escape airflows to estimate removal efficiency, utilizing the understanding that any aerosol leaving the ABCD with the escape airflow would not be removed effectively by the ABCD. This airflow-estimated removal efficiency is as in Eq 2:

$$\eta_{ABCD,airflow\;estimated}=1-\frac{Q_{escape}}{Q_{evac}+Q_{escape}}$$ (2)

where *η*_ABCD_ (-) is the airflow-estimated removal efficiency of the ABCD. In a real setting, this removal efficiency would parameterize how well the ABCD would effectively evacuate human-emitted aerosols so that they do not enter the bulk room air.

Finally, the simulation results from CFD runs (see next section) were used to compute a removal efficiency. This *CFD-estimated removal efficiency* was determined for each simulation run by utilizing knowledge of the mass of particles evacuated versus the mass escaping the ABCD, and normalizing the %-evacuated by the total of the %-evacuated and %-escaped:

$$\eta_{ABCD,CFD\;estimated}=1-\frac{{\%}_{escape}}{{\%}_{evac}+{\%}_{escape}}$$ (3)

This CFD-estimated removal efficiency is analogous to the airflow-estimated removal efficiency, and these metrics can be directly compared to each other to assess modeled-measured agreement.

### Computational fluid dynamics analysis

To extrapolate ABCD performance beyond the aerosol sizes evaluated by the physical experimentation, computational fluids dynamics (CFD) simulations were conducted using Siemen’s STAR-CCM+ software. CFD mathematically simulates a physical situation involving fluid flow by simultaneously numerically solving fluid equations with appropriate boundary conditions over a discretized (meshed) space. These CFD simulations replicated the experimental conditions where NaCl particles were emitted from a patient’s mouth for different opening conditions (i.e., potential pathways and flows for makeup air and particle escape). Only a subset of experiments were simulated using CFD, including experiments 1, 3, 4, and 5 at steady state conditions with a constant patient aerosol emission at the breathing flow rate of 10 L/min. These were all High flow experiments, but the seal of the ABCD varied from conditions of being tightly sealed to having open holes or no drape (see Fig 1A). The experiments utilized a continuous distribution of particle sizes using an atomizer, but the CFD simulations instead considered four discrete particle sizes of 0.1, 1, 5, and 10 μm in separate runs to investigate the containment ability over a possible range of emitted aerosol sizes. Furthermore, since the mode of the emitted experimental aerosol mass was near 0.1 μm, we were able to compare results of experiments 1, 3, 4, and 5 with the CFD results from the 0.1 μm runs, using the computed metrics described in Eqs 2 and 3 above for comparison. The full methodology for the CFD analysis is described in detail in the Supporting Information.

### Simulating impact of ABCD in log-risk-reduction framework

A range of plausible ABCD removal efficiencies informed by our experiments were used in simulations exploring the impact of different mitigation scenarios on exposures to healthcare providers. To do so, a two-zone model was used to estimate concentrations and inhaled dose of a near and far field provider in a room with the ABCD. Two-zone models [33] consist of coupled differential equations within a framework that treats concentrations in the personal air space (near field) immediately surrounding a person’s body as distinct from those in the bulk room air (far field). In our context, the near field provider represents a clinician directly near the ABCD (i.e., the “provider”), who is performing the AGP on a patient, while the far field provider represents a healthcare worker in the room but not directly near the ABCD (i.e., the “assistant”). Therefore, the near-field volume is that directly near the ABCD and including the head and breathing zone of the provider. The model assumes that any aerosol escaping the ABCD is emitted into the near field.

The near field concentration can be computed with Eq 4:

$$\frac{dC_{near}}{dt}=\frac{E(1-\eta_{ABCD})}{V_{near}}+\lambda_{near∙far}C_{far}-\lambda_{near∙far}C_{near}$$ (4)

where *C*_near_ and *C*_far_ (#/cm^3^ or μg/m^3^) is the near and far field concentrations, respectively; *E* (#/min or μg/min) is the emission rate of aerosols by the patient during the procedure; *V*_near_ (m^3^) is the near field volume; and *λ*_near-far_ (h^-1^) is the air exchange rate for near-far field volumes. In Eq 4, the first term is the emission into the near field volume, which is that emitted by the patient that escapes from the ABCD, and the second source term is that aerosol that is transported from the far field volume back into the near field volume. The loss term is that transported from the near field into the far field volume.

The far field concentration can be computed with Eq 5:

$$\frac{dC_{far}}{dt}=\lambda_{far∙near}C_{near}-\lambda_{far∙near}C_{far}-\lambda_{room}C_{far}$$ (5)

where *λ*_far-near_ (h^-1^) is the air exchange rate for far-near field volumes; and *λ*_room_ (h^-1^) is the air exchange rate of the clinical room. In Eq 5, the source term is that aerosol transported from the near field into the far field volume. The loss terms are that transported out of the far field back into the near field, or that transported out of the room with the room air exchange.

Using these near and far field concentrations, the inhaled dose for the provider in the near field, *D*_near_ (# or μg) can be computed with Eq 6:

$$D_{near}=Q_{br}(1-\eta_{m})\int_{t_{1}}^{t_{2}}C_{near}(t)dt$$ (6)

and the inhaled dose for the assistant in the far field, *D*_far_ (# or μg), can be computed with Eq 7:

$$D_{far}=Q_{br}(1-\eta_{m})\int_{t_{1}}^{t_{2}}C_{far}(t)dt$$ (7)

where *Q*_br_ (m^3^/min) is the average occupant breathing rate; *η*_m_ (-) is the mask efficiency for that worn by a healthcare provider in either the near or far fields; and *t*_1_ and *t*_2_ (min) are the start and end times of consideration for the dose calculation. In both expressions, the dose is computed as the product of the healthcare provider breathing rate, the penetration of aerosol through a protective mask, and the integral of the near or far field concentrations over a particular time horizon.

In the two-zone modeling analysis, the volumes were set at *V*_near_ = 1 m^3^ and *V*_far_ = 50 m^3^. The flow rate between the large and small volumes was 360 m^3^/h, so the *λ*_near-far_ = (360 m^3^/h ÷ 1 m^3^) = 360 h^-1^ and the *λ*_far-near_ = (360 m^3^/h ÷ 50 m^3^) = 7.2 h^-1^. An arbitrary patient emission was generated for 10 s and the *C*_near_ and *C*_far_ time-resolved concentrations were determined using coupled Eqs 4 and 5 over a time horizon of 10 min, using a numerical Euler method solution with a time-step of 0.0001 h = 0.36 s. By numerically integrating the time-resolved concentrations over the 10 min, the doses *D*_near_ and *D*_far_ were computed with Eqs 6 and 7, assuming a breathing rate of *Q*_br_ = 0.78 m^3^/h.

The impact of the ABCD with varying containment efficiencies was calculated in a parametric manner by contextualizing it within a set of protections including increasing the room air exchange rate *λ*_room_ and the healthcare provider mass efficiency *η*_m_. First, a baseline case was defined with *λ*_room_ = 1 h^-1^, no mask, and no use of the ABCD. Then, other cases were successively run in which driving variables were increased sequentially, so the simulations exhibited doses *D*_near_ and *D*_far_ that decreased. First, the room air exchange rate was raised to *λ*_room_ = 10 h^-1^; then, the mask efficiency was raised from *η*_m_ = 0 (no mask) to *η*_m_ = 0.6 (surgical mask) and 0.95 (N95 mask); and finally, the ABCD containment efficiency was raised from *η*_ABCD_ = 0 to *η*_ABCD_ = 0.5, 0.9, 0.99, and 0.999. For all these parametric simulation cases, the relative changes in *D*_near_ and *D*_far_ were computed by dividing the doses for each case by those of the baseline case, so that the use an arbitrary emission rate was warranted in a relative risk framework.

## Results

### Test aerosol behavior

Number and mass concentrations in select phases were averaged, so representative values could be established during key indicator periods during each experiment to demonstrated aspects of the ABCD performance. Averages included the measured background in the environmental chamber without aerosol generation (Phase 1), as well as in the environmental chamber due to escape from the ABCD during steady aerosol generation (Phases 4 and 6), in the environmental chamber due to escape from ABCD during simulated-coughing with aerosol generation (Phase 8), and in the ABCD itself due to direct emission from the mannequin mouth region (Phases 3, 5, and 7) (Fig 2D). The background aerosol concentration in the 1 m^3^ chamber was very low (always <12 #/cm^3^) over all eight experiments, so its impact was discarded in all analysis of the ABCD effectiveness.

Due to the strong aerosol emission directly into the ABCD, the measured number and mass concentrations in the ABCD were high (always >10^5^ #/cm^3^). However, the magnitude of the ABCD concentrations varied among experiments, because of differences in evacuation flow, relative separation between the ABCD and environmental chamber (i.e., how well sealed the ABCD was), and variability in aerosol emission rates by the generation system. To better constrain the reason for these variations, the number and mass emission rates of the aerosol were estimated (Fig 2A) using modeling and data-fitting techniques (see Materials and Methods, Supplementary Information). Emission rates were relatively constant across experiments, differing little by number and at most by about a factor of two by mass, giving confidence in the reproducibility of the experimental system. These ABCD concentrations are higher than that expected due to real aerosol emission from human expiratory activities, but these higher concentrations allow strong signals to be measured for the escape aerosol concentrations in the 1 m^3^ environmental chamber.

The concentration averages in the environmental chamber at steady state and during the simulated-coughing events demonstrate the general effectiveness of the ABCD at containing and removing generated aerosol (Fig 2D). For the steady state phases, the amount of escape aerosol in the environmental chamber generally depended on the ABCD test conditions. For High flow and Closed (or Hands) arm port and Neat drape conditions, almost no aerosol escaped (<6 #/cm^3^), but containment ability decreased as these stricter flow and sealing conditions were relaxed. The amount of aerosol escape followed similar trends for the simulated coughing. One point to note is there were larger differences between steady state and simulated coughing averages for more enclosed conditions. That is, the simulated coughing had more of an impact on the escape aerosol amount if the ABCD was well sealed and highly evacuated than if not (Fig 2D).

The size and mass distributions of the generated aerosol varied among experiments more in magnitude than by other distribution characteristics such as modal diameter or spread (Fig 2E and 2F). The aerosol size cutoff of >0.05 μm (with disregarded aerosol shown as a gray area on the plots) excluded more aerosol number than mass. Number and mass distributions were used to estimate the potential impact of aerosol deposition on the environmental chamber and ABCD concentrations, by using physical chamber dimensions and other aerosol and air properties to compute first order deposition loss rates for average particle conditions (see Materials and Methods) [32]. These deposition loss rates were estimated to be less than 0.1% of aerosol loss rates due to the ABCD evacuation (Fig 2G), so the impact of deposition on the steady state number and mass concentration was excluded from subsequent performance analysis.

### Aerosol containment performance

The average concentrations described above (Fig 2D) were used to compute the aerosol containment ability of the ABCD at the different experimental conditions. To do so, two different metrics were computed (see Materials and Methods). The first was the *containment fraction*, which is the fraction of aerosol number or mass in the ABCD compared to that in the total system of the ABCD and the 1 m^3^ environmental chamber. The second was the *airflow-estimated removal efficiency*, which is the evacuation flow rate normalized by the sum of the evacuation and escape flows (both of which were determined by the flow fitting process as described in Materials and Methods).

**Fig 3:**
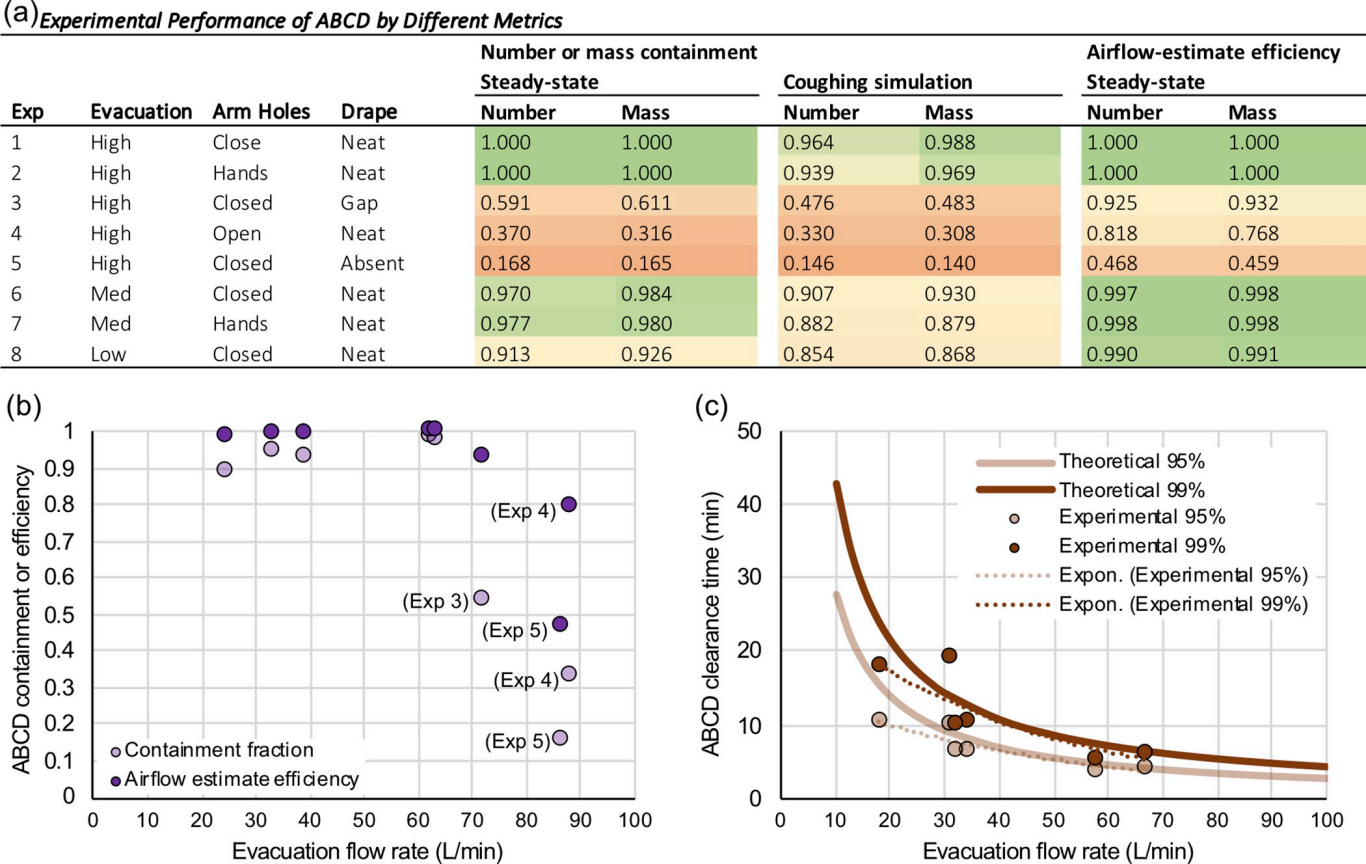
ABCD experimental performance. **(a)** Performance of ABCD during steady state phases for metrics of number or mass containment fraction [amount in ABCD / (amount in ABCD + amount in environmental chamber)] and airflow-estimated removal efficiency [ABCD evacuation flow rate / (ABCD evacuation flow rate + ABCD escape flow rate)], and during cough phase for containment fraction; **(b)** demonstration that reduced performance is a more function of ABCD seal properties, rather than evacuation flow rate; experiments with low containment or efficiency are noted on the plot; **(c)** demonstration of theoretical, experimental, and fitted ABCD clearance times (time for 95% or 99% cleared) as a function of evacuation flow rate, using data from number and mass results for Experiments 1, 6, and 8.

For each of the eight experiments, number and mass concentrations were individually used to compute mass- and number-based containment fractions and airflow-estimated removal efficiencies (Fig 3A). The containment fractions were calculated for two conditions—the first with average steady environmental chamber and ABCD concentrations, and the second with average simulated-coughing and ABCD concentrations. The airflow-estimated removal efficiency was only determined for the steady conditions (i.e., not coughing), because its calculation used fits computed with ABCD evacuation and escape flows with data taken during steady state Phases 3 to 7. However, the airflow-estimated removal should be similar during steady state and coughing scenarios, though it could be slightly and briefly influenced by the momentum of the coughing air flow.

The containment fractions are useful to demonstrate the performance of the ABCD at containing emitted particles within the ABCD versus escaping to the surrounding environment at a given moment in time. The airflow-estimated removal efficiency establishes how well the ABCD removes an emission over time, since it uses the possible flow paths (evacuation versus escape flows) to parameterize the expected ABCD performance. The removal efficiency is the superior metric from the perspective of emission control from a patient source, as it describes the eventual fate of emitted particles (i.e., probabilistically whether a particle gets evacuated or escapes the device), rather than being a snapshot of measured concentrations at a single timepoint (e.g., as with the containment fraction). These two performance metrics, of course, are highly correlated in their magnitude, because both are influenced by the relative amounts of evacuation versus escape flow rates.

According to these two performance metrics, the aerosol control ability of the ABCD varied widely by test condition, with each being very close to unity for well-sealed and strong evacuation conditions to less than one-half for poorly sealed conditions. To demonstrate how different test conditions affected ABCD performance, the containment fraction and airflow-estimated removal efficiencies were plotted as a function of the evacuation flow rate (Fig 3B). The plot demonstrates the interplay between evacuation flow and device seal in these experiments, showing that even low evacuation flow experiments with good ABCD sealing can result in effective containment and removal efficiency. Conversely, the plot shows that high evacuation flows are not enough to overcome poor sealing of the device and implies that good sealing was more important than high flow from a performance perspective in our tests.

Another important element regarding the ABCD performance is how quickly it evacuates aerosol once an emission episode has ceased, since ideally the ABCD would be left in place until it was appropriately cleared of any possible infectious aerosol. The time to clear a certain fraction of aerosol following an emission can be determined with first order decay theory using evacuation flow rate and ABCD volume. The time to clear 95% and 99% of the emission computed by theory was compared to the measured time for that same amount of aerosol to clear during decay Phase 9 of each experiment (Fig 3C). The theoretical and experimental results were very similar, especially at higher evacuation flow rates. The clearance time decreases with evacuation flow rate, and for this 0.1 m^3^ ABCD, evacuation flow rates of greater than ~40 L/min would correspond to 99% clearance of aerosol contaminants in ~10 min or less.

### CFD simulations

We experimentally tested ABCD efficacy using aerosols with diameter of 0.05 to 0.56 μm. This range could be generated by our experimental system, matched the range of measurement of the FMPS particle counter, and followed fluid streamlines and remained airborne for durations much longer than the residence time of air in the ABCD so airflow-related impacts could be isolated and deposition impacts could be neglected [34]. However, human expiratory activities generate particles of many sizes, including submicron and supermicron aerosol that remain suspended in air for long periods of time, as well as large droplets that settle quickly [4–6]. To understand how the ABCD might perform when challenged with emissions of larger aerosols, computational fluid dynamics (CFD) simulations were used.

**Fig 4:**
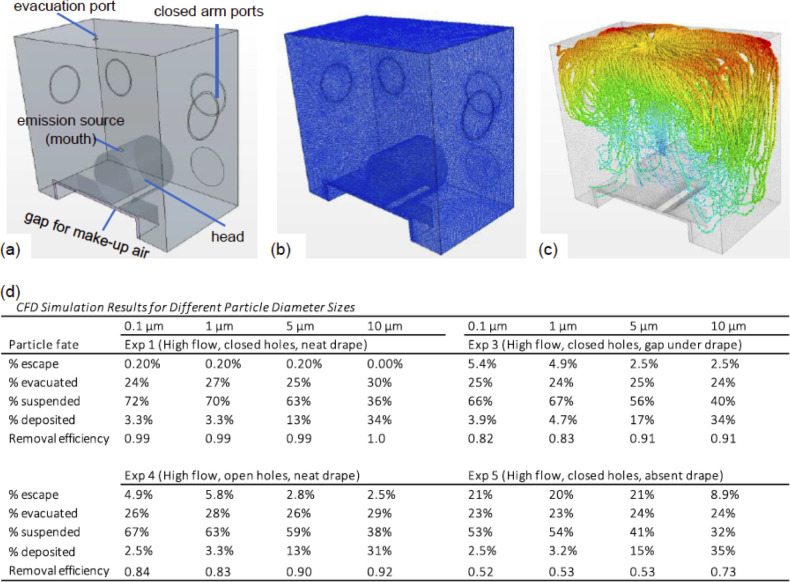
ABCD CFD simulations. **(a)** Simulation 3D geometry of ABCD details a supine patient exhaling particles at average breathing rate of 10 L/min. Patient head and shoulders are contained within the device; evacuation flow located along the top of the device and there is a gap for make-up air to flow in through the bottom of the device. Also shown are **(b)** CFD mesh with 800,000 cells; and **(c)** representative results of simulation for 0.1 μm particle size emissions where green/blue color indicates short duration (age of particle) after emission and orange/red color indicates long duration (red shows particles trapped suspended in corner of ABCD). **(d)** Particle fate in ABCD, where CFD simulations demonstrate increasing percentage of suspended particles and decreased percentage of deposited particles in the device as particle size decreases.

For the simulations, the real ABCD geometry was mimicked in the CFD software (Fig 4A) and discretized into a mesh with ~800,000 cells (Fig 4B). Experimental breath emission flows (10 L/min) and fitted particle emission rates (Fig 2A) were used to set CFD supply conditions, and fitted evacuation flow rates (Fig 2A) were used to set the CFD evacuation condition. Moreover, escape flows, which were also estimated with the fitting process (Fig 2A, see Materials and Methods), were set as the airflows between the inner ABCD air and the surrounding environment to calibrate the likelihood of particle escape in the CFD simulations. Particles were simulated using a Lagrangian method [35], whereby particles were emitted from the mouth and individual fates were determined as one of four options, including determining the fractional amounts escaping from, being evacuated from, remaining suspended in, or being deposited on a surface in the ABCD (Fig 4C). Particles of different sizes with a density of NaCl were simulated in distinct CFD runs, including those with diameters of 0.1, 1, 5, and 10 μm. The CFD simulations with 0.1 μm particles were intentionally similar to the sizes of the actual experimental particles (Fig 2F), so that good agreement in modeled and measured results would validate the CFD approach and support its extrapolation into the larger particle sizes.

To evaluate CFD model performance, the CFD results of evacuated and escaped aerosol mass were used to compute the *CFD-estimated removal efficiency*, by normalizing the %-evacuated by the total of the %-evacuated and %-escaped (Fig 4D, see Materials and Methods). This CFD-estimated removal efficiency can be directly compared to the airflow-estimated removal efficiency to assess model-measured agreement (Fig 3C). Four different experiments with conditions ranging from good to poor sealing were simulated using CFD. For the 0.1 μm aerosol cases, the comparisons of CFD-removal efficiencies to airflow-estimated removal efficiencies, respectively, for different experiments include: Experiment 1 (0.99 vs. 1.0 efficiency; conditions of High flow, Closed arm ports, Neat drape); Experiment 3 (0.82 vs. 0.93 efficiency; conditions of High flow, Closed arm ports, Gap drape); Experiment 4 (0.84 vs. 0.77 efficiency; conditions of High flow, Open arm ports, Neat drape); and Experiment 5 (0.52 vs. 0.45 efficiency; conditions of High flow, Closed arm ports, Absent drape). Due to the fact that we could only compare one experimental result to one CFD result per ABCD condition, it was not possible to compare the measurements versus simulations with a statistical approach. However, the CFD simulation results did agree well qualitatively with experimental results for the 0.1 μm aerosol cases.

Having compared qualitatively well to its experimental analog, the simulation metric of CFD-estimated removal efficiency can be used to demonstrate expected ABCD performance at larger aerosol sizes, and the provided analysis of aerosol fates (Fig 4D) also demonstrates trends in aerosol behavior. For the four simulated experimental conditions, the CFD-estimated removal efficiencies are similar for 0.1 and 1 μm diameter aerosols, but removal efficiencies increase at the larger diameters of 5 and 10 μm, suggesting that removal ability may be enhanced for emissions of larger aerosols. However, this increase in removal efficiency is because these larger diameter particles, with their much larger mass, deposit onto surfaces inside the ABCD at higher rates than smaller particles. Indeed, the aerosol fates demonstrate that the %-evacuated remains relatively constant for the larger diameter particles, but that the %-escaped and %-suspended decreased while the %-deposited increased compared to smaller diameter aerosols. It should be noted that this larger particle deposition inside the ABCD supports the need for good cleaning of the ABCD between patients, or the need for a similar single-use device. These aerosols could also deposit onto the patient body or garments, also suggesting use of a single use garment during the procedure.

### Log-risk-reduction framework

**Fig 5:**
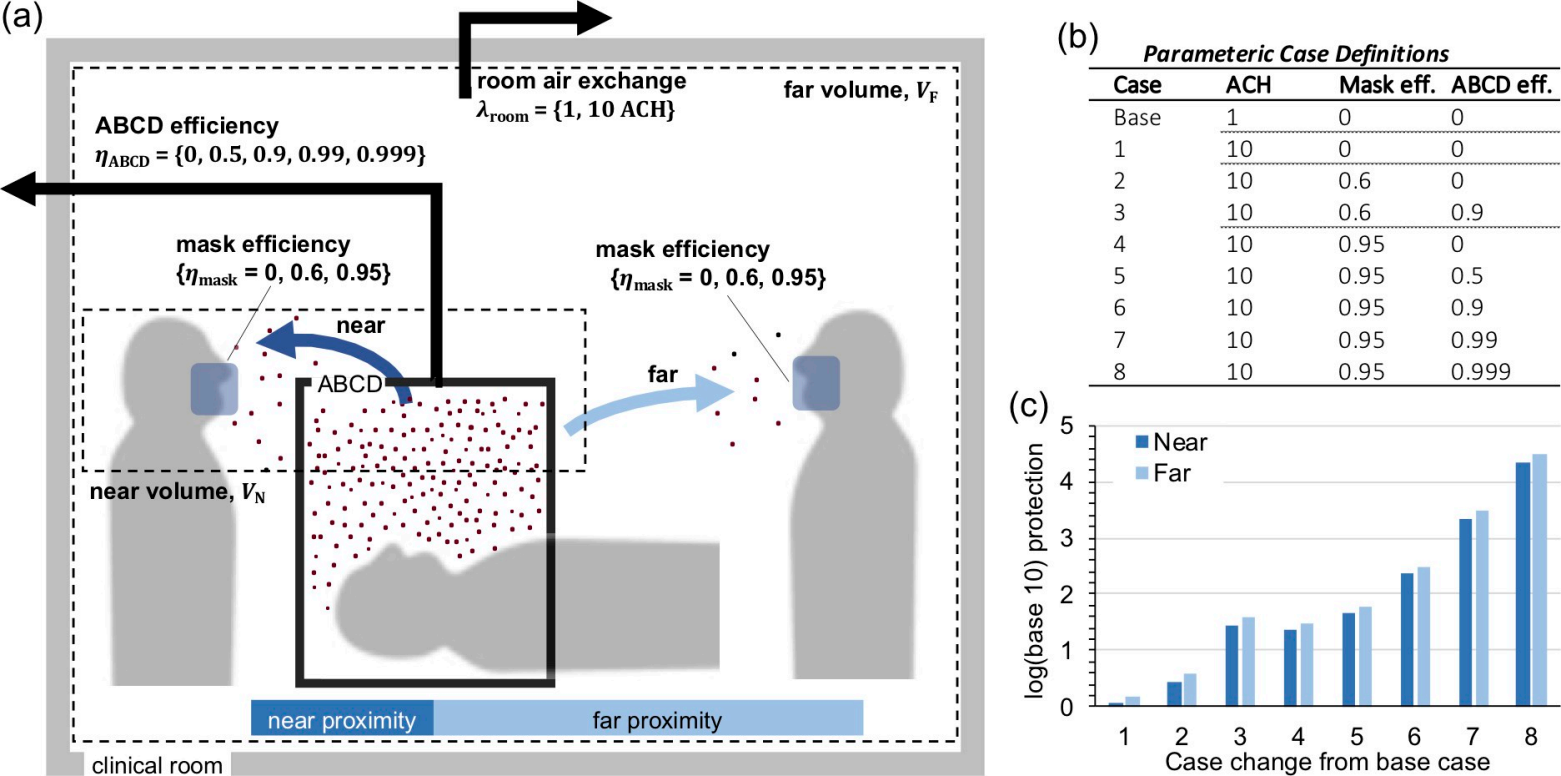
Log-risk-reduction framework. **(a)** Schematic of framework used to simulate impact of ABCD in a hypothetical clinical setting, using a two-zone well-mixed model. Patient emitted aerosols that escape the ABCD enter the near volume, in which exists a near proximity clinical worker, and then aerosols are transported from the near volume into the far volume, in which exists a far proximity clinical worker. **(b)** Table of nine simulated cases with different levels of protections, which started at a low protection base case and then increased protections to the highest simulated combination of protections. **(c)** Log_10_ protection according to inhaled dose of near and far proximity clinical workers for each protective case normalized by the base case (i.e., co-log_10_ of dose ratios).

Using the range of demonstrated removal efficiencies as a guide, we simulated the impact of the ABCD in a clinical setting using a log-risk-reduction framework (Fig 5A). Protective capacity was computed using integrated dose for a discrete emission emitted from a patient, considering dose reductions due to different combinations of potential mitigation strategies. Since healthcare providers near and far from the patient can have different exposures, dose was computed for each using a two-zone modeling framework. Different combinations of mitigation strategies that would influence dose (and so airborne transmission) were evaluated in a progressive approach, relative to risk for a baseline with no protections, and layering on mitigations considering room air changes per hour (ACH), facemask efficiency, and ABCD efficiency (Fig 5A). It is called a log-risk-reduction framework because, depending on the key variable strengths, the layered protection can achieve total protective efficiencies on order of 0.9 (1-log), 0.99 (2-log), 0.999 (3-log), or even 0.9999 (4-log) or more for optimal combinations of the variables (high evacuation and effective sealing of the ABCD, high ACH, and N95 facemasks worn by proceduralists).

This framework allows us to contextualize and understand how an aerosol containment device like the ABCD can be integrated into airborne protection methods involving PPE and environmental controls to influence overall risk of transmission, over a considered set of combinations (Fig 5B). The results (Fig 5C) demonstrate that use of the ABCD functioning at 90% efficiency together with a typical surgical mask of approximately 60% efficiency [36] reduces particle exposure by about 1-log more than the surgical mask alone (comparing case 2 and 3). This estimated protection is slightly better than the estimated protection provided by an N95 (95% efficiency mask) alone (comparing case 3 and 4) and has the added benefit of reducing potential room contamination. These results suggest that in the absence of N95 masks, the ABCD together with a standard surgical mask could provide airborne protection on the same order of magnitude as an N95 mask, which could be a strong approach in times of N95 shortage.

## Discussion

Airway procedure protective devices that use suction have been described elsewhere [28, 37] and have a range of access ports and methods of creating a sealed enclosure around a patient [24–28]. Some devices are partially open, whereas others create a tighter seal around a patient. Our test results with the ABCD indicate that open access ports as well as lack of or inefficient seal over the patient torso strongly reduced device removal efficiency and performance (Fig 3B). Recent evidence suggests that protective barrier devices can even increase airborne particle concentrations and risk of aerosol exposure [25]. That is, an open or poorly sealed protective device could lead to a situation where aerosols are concentrated within the device prior to escape into the room, which could then increase local aerosol concentrations near a health care providing device user. The need for a tight seal with a containment device needs to be balanced against the clinical utility of a device where patient access or user mobility still allows for safe completion of a medical airway procedure.

For these types of devices, evacuation airflow is required under the current FDA EUA [38]. Evacuation flow rates can vary widely, and a minimum standard flow rate has not been established. The amount of time a device should remain in place after use with suction operational to clear contaminated device air has not been fully explored [39]. Our results suggest that suction flow rates with devices similar in size to the ABCD should be ≥40 L/min. This evacuation flow rate will ensure >90% removal of any infectious aerosol and will also reduce the amount of time required to clear contaminated air from the device after an airway is secured to ≤10 minutes (Fig 3C). Aerosol clearance time from room air depends on the particle size and number of room air changes per hour (ACH). There are minimum standards for air change rates in the U.S., with hospital-based clinic rooms needing 6 ACH, negative pressure rooms 12 ACH, and operating rooms 15 ACH [40]. The actual rate of aerosol clearance from the local vicinity of the healthcare providers additionally depends on the location of supply diffusers and return grills, patient respiratory rate and tidal volumes, and temperature differential between room air and patient breath. Furthermore, based on the CFD simulations (Fig 4), larger aerosols (with particle diameters >5–10 μm) would tend to deposit and contaminate the device. This occurrence highlights an important consideration that devices contaminated with infectious particles need rigorous cleaning protocols to reduce risks of re-aerosolization and secondary contamination. Another option would be to use disposable single-use devices (or garments) rather than re-useable devices.

In addition to reducing airborne transmission of SARS-CoV-2, there is a long-term need to re-think the approach to airborne pathogen exposure that incorporates layered risk reduction involving aspects such as vaccines (if available), rapid point-of-care testing (if available), enhanced PPE, improved air exchange and airflow management, and aerosol protective enclosure devices. Hospitals have a limited number of rooms with very high air exchange rates or negative pressure rooms that can minimize aerosol spread. The best available PPE at this time would be a power air purifying respirator (PAPR) or N95 with face shield for all involved staff, but these would not keep the room itself free of contamination. The ABCD can also be used in conjunction with an N95 facemask to provide additional, layered protection. Using the log-risk-reduction framework, we estimate that the ABCD can provide an additional 1-log (ABCD operating at 90% efficiency) to 3-log (ABCD operating at 99.9% efficiency) reduction in airborne particle exposure compared to using an N95 facemask alone (Fig 5C, comparing cases 6, 7, and 8 to case 4). This substantial reduction in airborne particle exposure may be a helpful adjunct to further reduce exposure risk particularly with emerging or future variants of SARS-CoV-2 or for protection against existing or emerging highly infectious airborne pathogens.

Medical grade masks and respirators have established quantitative standards for rating the efficiency of aerosol filtration. There are currently no standards for rating or assessing the efficacy of airborne protective containment devices. Studies in these novel protective devices have utilized particle measurement in open systems, evaluation of airflow patterns (e.g., smoke evacuation), or simulations to examine device function [41, 42]. However, it is difficult to extrapolate device efficiencies from these uncontrolled test conditions or scenarios. The evaluation methodology presented herein involves testing the device in a larger closed environmental chamber with particle-free air, which allowed us to accurately quantify aerosol concentrations inside and outside the device. By determining removal metrics using the experimental data, we were able to expand our analyses to include simulations using CFD over a range of aerosol sizes and simulations of clinical scenarios to perform parametric analyses to probe device efficacy for different use cases in the context of other mitigation measures. We suggest that our methodology can serve as the basis for rating the efficacy of these airborne containment devices. We also proffer that a similar framework to our log-risk-reduction framework can be used to understand how these devices interact as part of a comprehensive layered protection against airborne infectious diseases.

In summary, this work demonstrated with laboratory testing and CFD simulations that the ABCD was effective in reducing aerosol escape and would reduce clinical exposure. We utilized steady state and simulated coughing aerosol emissions in the ABCD, along with concentration measurements and fitted evacuation and escape flow rate conditions, to determine the containment fraction and airflow-estimated removal efficiency of the ABCD. We assessed the efficacy of the ABCD under different evacuation and sealing conditions that we anticipate will mimic real-world use scenarios and established that the device effectiveness depended on evacuation rates and, more importantly, on the overall seal of the device. The efficacy was assessed using two different but related metrics, and we posit that removal efficiency is a more useful metric than the containment fraction, since removal efficiency indicates the eventual probabilistic fate of any particles emitted by a patient.

## Supporting information

S1 Fig Airflow schematic. (a) Schematic of airflows during the decay portion of each experiment (Phase 9), during which aerosol generation was off and particle sampling was from within ABCD. (b) Schematic of airflows during steady state portions of each experiment (Phases 4 and 6), during which aerosol generation was on and particle sampling was from within the 1 m^3^ environmental chamber.
(TIFF)

S1 File (DOCX)

S1 Data (XLSX)
