# Supplementary figures and images for: Design and quantitative evaluation of ‘Aerosol Bio-Containment Device (ABCD)’ for reducing aerosol exposure during infectious aerosol-generating events
Source: PLoS One. 2023 Jan 6;18(1):e0272716. doi: 10.1371/journal.pone.0272716 (PMC9821519; doi:10.1371/journal.pone.0272716)

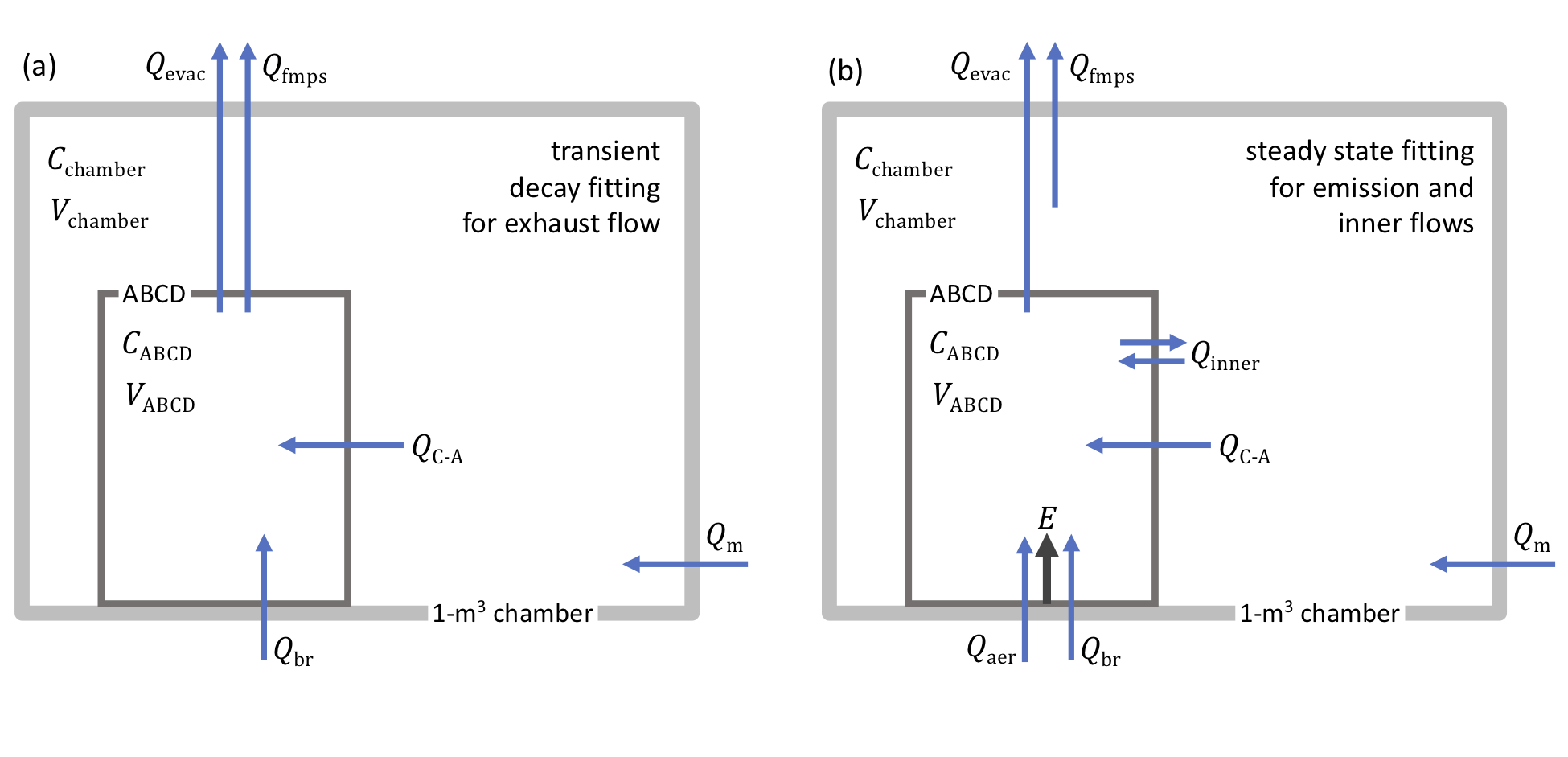

Supplement: S1 Fig — (a) Schematic of airflows during the decay portion of each experiment (Phase 9), during which aerosol generation was off and particle sampling was from within ABCD. (b) Schematic of airflows during steady state portions of each experiment (Phases 4 and 6), during which aerosol generation was on and particle sampling was from within the 1 m3 environmental chamber. (TIFF) [file pone.0272716.s001.tiff]
