# Supplementary material for: Design and quantitative evaluation of ‘Aerosol Bio-Containment Device (ABCD)’ for reducing aerosol exposure during infectious aerosol-generating events
Source: PLoS One. 2023 Jan 6;18(1):e0272716. doi: 10.1371/journal.pone.0272716 (PMC9821519; doi:10.1371/journal.pone.0272716)
Supplement: S1 File — (DOCX) [file pone.0272716.s002.docx]

**SUPPORTING INFORMATION FOR:**

**Design and quantitative evaluation of ‘Aerosol Bio-Containment Device (ABCD)’ for reducing aerosol exposure during infectious aerosol-generating events**

Michael S. Waring,^1^* L. James Lo,^1^ Michael A. Kohanski,^2^ Elizabeth Kahle,^3^ Ian M. Marcus,^1^ Heather Smith,^4^ Kara L. Spiller,^3^ Sharon L. Walker^1^

**Corresponding author*:

Michael Waring, [msw59@drexel.edu](mailto:msw59@drexel.edu)

*Affiliations*:

**^1^**Department of Civil, Architectural and Environmental Engineering, Drexel University, Philadelphia, PA, USA

**^2^**Department of Otorhinolaryngology-Head and Neck Surgery, Division of Rhinology, University of Pennsylvania, Perelman School of Medicine, Philadelphia, PA, USA

**^3^**School of Biomedical Engineering, Science, and Health Systems, Drexel University, Philadelphia, PA, USA

**^4^**Life Sciences Department, Riverside City College, Riverside, CA, USA

This Supporting Information file contains methodological information in these sections:

Section S1. Estimating flow rates and aerosol emission rates

Section S2. Computational fluid dynamics (CFD) analysis

**Section S1. Estimating flow rates and aerosol emission rates**

Certain parameters were not directly measured in the experiments but were instead computing using two similar but separate fitting process with data from different experimental phases, including evacuation and ABCD escape flow rates and aerosol emission rates. These parameters were used to constrain performance of the ABCD (i.e., calculate containment ability), as well as to set boundary conditions for computational fluid dynamics (CFD) simulations.

***Estimation of evacuation flow rate*.** The first fitting process determined evacuation flow rates. Vacuum pumps were used to provide evacuation airflows for the ABCD between ~20 and 90 L/min in the test system, depending on the desired experimental condition (i.e., low, medium, or high evacuation, which corresponded to use of one, two, or three pumps). The laboratory did not have a means to accurately measure airflows at these higher rates, so evacuation flow rates were computed using aerosol concentration data from the Phase 9 transient decay portion of each experiment. Fig S1a demonstrates the operational state of the chamber flows and sampling during Phase 9 of all experiments. During the Phase 9 decay portion, sampling occurred from inside the ABCD and the aerosol generation system was turned off, so airflows out of the ABCD were evacuation (*Q*_evac_, L/min) and FMPS (*Q*_fmps_, L/min) only, while airflows into the ABCD were the supplemental breath airflow (*Q*_br_, L/min) and airflow from the 1 m^3^ environmental chamber into the ABCD during the decay phase (*Q*_C-A,d_, L/min). Also, the 1 m^3^ environmental chamber had makeup airflow (*Q*_m_, L/min).

**Fig S1. Airflow schematic.** (a) Schematic of airflows during the decay portion of each experiment (Phase 9), during which aerosol generation was off and particle sampling was from within ABCD. (b) Schematic of airflows during steady state portions of each experiment (Phases 4 and 6), during which aerosol generation was on and particle sampling was from within the 1 m^3^ environmental chamber.

For this first fitting process, concentration balances on particle number and mass concentrations were individually used to constrain *Q*_evac_ for each experiment using results from the decay period during Phase 9. To do so, a value of *Q*_evac_ was determined by minimizing the sum of squared difference between measured and modeled ABCD concentrations. The ABCD concentration was modeled using a standard concentration balance that treats the ABCD and 1 m^3^ environmental chamber each as well-mixed continuous mixed flow reactors, where the two chambers are connected by airflows, as in:

| $\frac{dC_{\mathrm{ABCD}}}{dt}=\left( \frac{Q_{C-A,d}}{V_{\mathrm{ABCD}}} \right)C_{\mathrm{chamber}}-\left( \frac{Q_{\mathrm{evac}}+Q_{\mathrm{fmps}}}{V_{\mathrm{ABCD}}} \right)C_{\mathrm{ABCD}}$ | (S1) |
| --- | --- |

where variables not yet defined are *t* (min) is time; *C*_ABCD_ and *C*_chamber_ (#/cm^3^ or μg/m^3^) are number or mass aerosol concentrations in the ABCD and 1 m^3^ chamber, respectively; and *V*_ABCD_ (m^3^) is the ABCD volume. In Equation S1, the positive term is the source of aerosol in the ABCD from that of airflow into the ABCD from the 1 m^3^ environmental chamber carrying any aerosol that escaped from the ABCD into the 1 m^3^ environmental chamber during previous experimental phases, and the negative term is the loss of aerosol from the ABCD due to evacuation and FMPS sampling airflows.

In Equation S1, some terms were directly known or actively measured during Phase 9, including *C*_ABCD_, *V*_ABCD_, *Q*_fmps_, and *Q*_br_. The primary airflow from the 1 m^3^ chamber into the ABCD, *Q*_C-A,d_ (L/min), is unique to the decay condition and is determined by balancing other airflows in the chamber, so *Q*_C-A,d_ = *Q*_evac_ + *Q*_fmps_ – *Q*_br_. However, Equation S1 also requires knowing the concentration in the 1 m^3^ chamber, *C*_chamber_, which was not directly measured because sampling occurred from inside the ABCD during decay Phase 9. Therefore, the *C*_chamber_ was estimated during each time step of Phase 9 using a decay balance with an initial condition of *C*_chamber_ as that measured immediately prior to the start of Phase 9, with:

| $\frac{dC_{\mathrm{chamber}}}{dt}=\left( \frac{Q_{C-A,d}}{V_{\mathrm{chamber}}} \right)C_{\mathrm{chamber}}$ | (S2) |
| --- | --- |

where *V*_chamber_ (m^3^) is the 1 m^3^ chamber volume. Using Equations S1 and S2, the only unknown parameter during decay Phase 9 was *Q*_evac_, so it could be fit for each experiment using the estimation procedure.

***Estimation of escape flow and aerosol emission rates*.** Once *Q*_evac_ was determined for each experiment using particle number and mass concentrations, the second fitting process used a similar but separate procedure to estimate two other parameters, by considering the experimental condition of Phases 4 and 6 when the aerosol emission system was operating and steady-state particle escape from the ABCD was measured in the 1 m^3^ environmental chamber, as shown in Fig S1b. The estimated parameters in this case were the number or mass emission rate, *E* (#/min or μg/min) and the *Q*_inner_ (L/min) airflow, which parameterizes two-way airflow between the ABCD and 1 m^3^ environmental chamber during the experiments. This parameter *Q*_inner_ increases in experiments when more aerosol escapes from the ABCD. It is a simplification of a more complex physical situation that is intended to describe inter-chamber airflow from an average perspective. In a real situation, this two-way inner airflow would parameterize the ‘escape’ airflow from the ABCD, and it is used as such in the next section as *Q*_escape_ (L/min).

For the condition during Phases 4 and 6, the concentration balance for the 1 m^3^ environmental chamber concentration, *C*_chamber_, is as shown in Equation S3:

| $\frac{dC_{\mathrm{chamber}}}{dt}=\frac{Q_{\mathrm{inner}}}{V_{\mathrm{chamber}}}C_{\mathrm{ABCD}}-\left( \frac{Q_{C-A,s}+Q_{\mathrm{fmps}}+Q_{\mathrm{inner}}}{V_{\mathrm{chamber}}} \right)C_{\mathrm{chamber}}$ | (S3) |
| --- | --- |

In Equation S3, the first term is the source of the non-retained aerosol from the ABCD into the 1 m^3^ chamber with the two-way inner airflow, and the second term is the loss of the 1 m^3^ chamber aerosol with the 1 m^3^ chamber-into-ABCD, FMPS sampling, and two-way inner airflows. In this experimental condition, the primary airflow from the 1 m^3^ chamber-into-ABCD, *Q*_C-A,s_ (L/min), is different at steady-state than during the decay condition, and it is again determined by balancing the inflows and outflows in the ABCD, so *Q*_C-A,s_ = *Q*_evac_ – *Q*_br_  – *Q*_aer_.

Furthermore, Equation S3 includes the parameter of the ABCD concentration, *C*_ABCD_, which may be described with a mass balance as in Equation S4:

| $\frac{dC_{\mathrm{ABCD}}}{dt}=\frac{E}{V_{\mathrm{ABCD}}}+\left( \frac{Q_{C-A,s}+Q_{\mathrm{inner}}}{V_{\mathrm{ABCD}}} \right)C_{\mathrm{chamber}}-\left( \frac{Q_{\mathrm{evac}}+Q_{\mathrm{inner}}}{V_{\mathrm{ABCD}}} \right)C_{\mathrm{ABCD}}$ | (S4) |
| --- | --- |

In Equation S4, the first term is the source from the direct emission of the aerosol into the ABCD from the mannequin, the second term is the source from the 1 m^3^ chamber of non-retained aerosol back into the ABCD with the 1 m^3^ chamber-to-ABCD airflow and the two-way inner airflow, and the third term is loss of aerosol in the ABCD to the evacuation flow or the two-way inner flow.

Since the experimental condition during Phases 4 and 6 is at steady-state, the 1 m^3^ environmental chamber and ABCD conditions and concentrations can be assumed to be unchanging in time, so Equations S3 and S4 can be recast in their steady-state forms, respectively, as Equation S5:

| $C_{chamber,ss}=\frac{Q_{\mathrm{inner}}C_{ABCD,ss}}{Q_{C-A,s}+Q_{\mathrm{fmps}}+Q_{\mathrm{inner}}}$ | (S5) |
| --- | --- |

and as in Equation S6:

| $C_{ABCD,ss}=\frac{E+\left( Q_{C-A,s}+Q_{\mathrm{inner}} \right)C_{chamber,ss}}{Q_{\mathrm{evac}}+Q_{\mathrm{inner}}}$ | (S6) |
| --- | --- |

where *C*_chamber,ss_ and *C*_ABCD,ss_ denote the steady-state concentrations in the 1 m^3^ chamber and ABCD, respectively. Using the steady-state concentrations during this condition, the emission rate, *E*, and two-way inner airflow, *Q*_inner_, were fit by minimizing the total sum-of-squared difference between the measured and modeled 1 m^3^ and ABCD chamber concentrations. Since the ABCD concentrations were not actually measured during Phases 4 and 6, the concentrations measured at the end of Phases 3 and 5 were used as surrogate measured ABCD concentrations for this process.

One last point to note relates to the volumes used in these fitting methods, *V*_chamber_ and *V*_ABCD_, in Equations S1 to S6. These volumes are not the nominal environmental chamber (1 m^3^) or ABCD (0.1 m^3^) volumes since those are the volumes without any material inside. Instead, these *V*_chamber_ and *V*_ABCD_ are the actual air volumes in each space, accounting for the various materials within each, including the ABCD and mannequin body in the 1m^3^ chamber and the mannequin head and shoulders in the ABCD. The volumes were estimated using known dimensions and volume displacement methods for the mannequin elements, resulting in best estimates of *V*_chamber_ = 0.87 m^3^ and *V*_ABCD_ = 0.093 m^3^.

**Section S2. Computational fluid dynamics (CFD) analysis**

To extrapolate ABCD performance beyond the aerosol sizes evaluated by the physical experimentation, CFD simulations were conducted using Siemen’s STAR-CCM+ software. These CFD simulations replicated the experimental conditions where solid NaCl particles were injected from a patient’s mouth and various opening conditions (i.e., potential pathways for makeup air and particle escape) were modeled based on the experimental matrix (see Fig 2a in main paper).

***Governing equations for CFD.*** CFD simulations include equations for modeling the movement of the fluid (air) and the emitted aerosol particles. Typical Reynolds-Averaged Navier–Stokes (RANS) equations models were used simulate and establish the fluid regime. A realizable two-layer k-epsilon turbulence model was used in these simulations with the default constants. A Lagrangian multiphase model in STAR-CCM+ was chosen to simulate solid NaCl particles suspended in airflow fields and allow tracking of their movement within the simulation domain. Lagrangian particle tracking uses and individually follows representative particle “packets” of a given mass to simulate understand particle movement as it less computationally burdensome. We did not include thermal heat from the human element, since the mannequin was at room temperature, so a segregated fluid flow model was chosen. Each simulation was conducted at steady state conditions, as the key comparison variable (airflow-estimated removal efficiency, see main paper) was calculated during steady state experimental phases. Further assumptions included that the flow is incompressible (true for indoor air under normal circumstances) and that during steady state there would not be considerable transformation of particles (e.g., evaporation) before leaving the domain.

***Model geometry and mesh.*** The CFD model geometry includes a representation of the ABCD with a simplified patient head and top of shoulders placed inside the ABCD to mimic experimental conditions (see Fig 4a in main paper). The patient’s head was simplified to a cylinder for ease of simulation and because facial features are unlikely to impact exhaled flow. The simulated open mouth (a small 5 cm by 2 cm rectangular surface) expels air (breath) with a particle emission. The CFD domain was discretized by automatically generated polyhedral cells with base size of 1 cm, and finer mesh was used near the patient body, inlet, and outlet flows. A low surface and volume growth value of 1.1 was used to create a smooth transition between fine and coarse cells. Grid size was chosen based on a grid independence study of three mesh configurations with 100k, 800k, and 1.5 million cells where the 800k cells version was selected due to the balanced accuracy and computation time (see Fig 4b).

***Boundary conditions and particle definition.*** For these simulations, four boundary conditions were simulated: the 1) suction used to evacuate the particles, 2) exhaled air and particle emission coming from the patient mouth, 3) openings (arm holes or opening around the patient torso) which can either provide make-up air into the ABCD or allow air and particle can escape from ABCD, and 4) surfaces inside the ABCD onto which particles can deposit. The aerosol emission from the patient’s mouth had to provide an emitted mixture of particles and air, so this emission was modeled as a CFD mass flow fluid inlet in conjunction with a particle injection condition. Specific airflow rates and particle mass emission were set to match the experiment conditions. While the experiment produced a distribution of particle sizes using an atomizer, the CFD simulations modelled four distinct particle sizes, 0.1, 1, 5, and 10 μm, in order to investigate the transport behavior due to the possible range of emitted aerosol sizes.

***Convergence.*** A CFD model is a numerical model that must converge in order to reach a solution. For the steady-state convergence requirement, we examined both the simulation residuals (differences between iterations) and variance of particle mass flow. In terms of simulation residuals, both continuity and momentum solutions reached below 10^-5^, a typical acceptance value for flow convergence. The turbulence residual was higher at 10^-3^ due to part of the highly turbulent flows near the suction outlet on the top of the ABCD. For the particle mass flow variance, we monitored particle mass outflow and deposition on the walls of ABCD, ensuring the change in mass flow and deposition rate did not vary over 5% over iterations. With these requirements, the solution converged after between 1500 to 3000 iterations based on the differences in simulated geometry (different openings) and flowrates among scenarios.

***Result calculation.*** The CFD analysis allows an understanding of the particle fate during the use of the ABCD, using mass flow and accumulation inside the model domain. The injected particles (from the mouth) can have one of four potential fates: 1) evacuate from ABCD via the suction port; 2) escape from the ABCD openings (arm holes or gap around the patient); 3) deposit onto a surface in the ABCD; 4) be suspended in the air. With a mass balance for particles, these fates can simply be represented by:

| $M_{\mathrm{injected}}=M_{\mathrm{evacuated}}+M_{\mathrm{escaped}}+M_{\mathrm{deposited}}+M_{\mathrm{suspended}}$ | (S7) |
| --- | --- |

Since particle injection mass was a set boundary condition, and particle mass flux can be quantified at the suction port, at escape openings of arm holes and gaps, and at surfaces inside the ABCD, the mass balance can be used to calculate the mass of particles suspended in the air. Using known mass outcomes, we can represent the relative fates as percentages.
